# Knowledge on First Aid Management of Epistaxis among Medical Students of a Medical College: A Descriptive Cross-sectional Study

**DOI:** 10.31729/jnma.8580

**Published:** 2024-05-31

**Authors:** Shiva Bhushan Pandit, Bipin Koirala, Rajeev Kumar Shah, Yoveen Kumar Yadav

**Affiliations:** 1 Department of Otorhinolaryngology, Head and Neck Surgery, Birat Medical College Teaching Hospital, Tankisinuwari, Morang, Nepal; 2 Hope International College, Kathmandu, Nepal

**Keywords:** *epistaxis*, *first aid*, *medical students*, *otorhinolaryngology*

## Abstract

**Introduction:**

Epistaxis, a global Otorhinolaryngology emergency, often requires hospital admission, posing health concerns for all ages. Evaluating medical students' knowledge helps identify strengths and areas for improvement, ensuring future healthcare providers are well-prepared. Our study aimed to assess knowledge on first add management of epistaxis among medical students at a medical college.

**Methods:**

This descriptive cross-sectional study was among medical students of Birat Medical College and Teaching Hospital after ethical approval was obtained. Data was collected electronically using google form from 109 medical students from 15 January 2024 to 15 March 2024.

**Results:**

The mean age of students was 20.71±1.44 years. The mean knowledge score of first aid management of epistaxis was 11.33± 5.24. Out of all students, 68 (62.38%) students had above-average knowledge on first aid management of epistaxis.

**Conclusions:**

The study emphasizes the varied knowledge levels among first and second-semester medical students regarding epistaxis. While more than half demonstrated above-average understanding, targeted educational interventions are warranted.

## INTRODUCTION

Epistaxis, a common Otorhinolaryngology emergency worldwide, often requires hospitalization, posing significant health risks across age groups. Globally, about 60% of individuals experience epistaxis in their lifetime, with 10% necessitating severe medical intervention.^1^ Recurrent or severe episodes of epistaxis can lead to complications such as anemia, psychological distress, and disruptions in daily activities.^2^

Prompt and accurate intervention is crucial to prevent complications, making it imperative to assess the knowledge on its first aid management. Studies conducted among health care providers and the general population showed lack of knowledge on first aid management of epistaxis.^3,4^ Evaluating medical students' knowledge aids in identifying both strengths and areas for improvement in students' skills.

Thus, we aimed to assess knowledge on first aid management of epistaxis among medical students at a medical college at eastern Nepal.

## METHODS

This descriptive cross-sectional study was conducted among medical students of Birat Medical College Teaching Hospital. Ethical approval was taken from the institutional review committee of Birat Medical College Teaching Hospital prior to conducting research (Reference number: IRC-PA-368/2024). Informed consent was obtained after participants were informed about the objective of the research. MBBS students of first semester and second semester of batch 2022 and 2023 willing to participate in the study were enrolled for the study. Students who refused participation were excluded from the study.

A total of 200 MBBS students were invited to be enrolled to the study of which 109 students participated in the study.

Data was collected using a self-administered questionnaire on epistaxis from 15 January 2024 to 15 March 2024. The first part of the questionnaire included participants' age, gender, batch, previous or recent history of epistaxis and the management technique. The second part of the questionnaires assessed knowledge on causes, most common sites involved and its first aid management.

Participants correctly responding to the knowledge related questionnaires on epistaxis were scored 1 and those with incorrect answers were scored 0. Total knowledge related questionnaires were 30. The collected data were transferred in Microsoft excel and analysed by SPSS software. Participants obtaining above the mean score were considered having aboveaverage knowledge and participants obtaining equal to or below mean score were considered below-average knowledge.

## RESULTS

The total of 109 students participated in the study. of which there were 54 (49.54%) female and 55 (50.46%) male. The mean age of students was 20.70±1.44 years.

Out of all participants, 72 (66.05%) stated that they had experienced the occurrence of epistaxis. Among 72 (66.05%), 40 (55.55%) had self-suffered from epistaxis while 18 (25%) had seen among family members and 14 (19.44%) among the relatives. Among them 6 (8.33%) had correctly managed epistaxis. After home managemenet 17 (23.61%) participants had consulted doctor (Table 1).

The mean knowledge score of students was 11.33± 5.247. Out of 109 students, 68 (62.38%) students had above average knowledge and 41 (37.62%) on first aid management of epistaxis.

**Table 1:** Past experiences and home management of epistaxis (n= 109).

Variables	n (%)
Ever experienced epistaxis	72 (66.05)
Self	40 (55.55)
Family members	18 (25)
Relatives	14 (19.44)
**Home management of epistaxis when suffered/ experienced (n=72)**	
Pinch soft part, lean forward for sometimes (correct practice)	6 (8.33)
**Incorrect Practice (n=66)**	
Pinch soft part of nose only	5 (7.57)
Pinch soft part of nose, lean backward for sometimes	23 (34.85)
Lean backward for sometimes	30 (45.45)
Did nothing at home and directly visit doctor as soon as bleeding start	1 (1.51)
Lean forward for sometimes without pinching	7 (10.61)

Out of all participants, 82 (75.22%) students correctly responded that the inner nose is the source of epistaxis, 66 (60.55%) students correctly responded that using blood thinning agents causes epistaxis while 23 (20.1%) correctly responded that pregnancy can also cause epistaxis, 105 (96.33%) correctly responded that epistaxis can occur at any age and sex group, 7 (6.42%) students correctly mentioned the primary management of epistaxis. Students correctly responding to the measures to be taken for maintaining breathing during nose pinching and cartilage part of nose to pinch while breathing were 56 (51.4%) each (Table 2).

**Table 2:** Knowledge of students on different components of epistaxis and its first aid management (n=109)

Characteristics	n (%)
**Knowledge on source of bleeding**	
Inner nose (correct response)	82 (75.23)
**Cause of epistaxis (correct responses)**	
Use of blood thinning agents (aspirin, warfarin)	66 (60.55)
Pregnancy	23 (21.10)
Epistaxis can occur at any age and among any sex (correct response)	105 (96.33)
**Knowledge on most common type of epistaxis**	
Anterior nose bleeding (correct response)	71 (65.14)
**Knowledge on most serious types of epistaxis**	
Posterior nasal bleeding (correct response)	72 (66.05)
**Knowledge on Primary steps in management of epistaxis**	
Sitting and leaning head slightly forward, Pinching nose with thumb and fore finger, Maintain airway (correct responses)	7 (6.42)
**Knowledge on average time for pinching nose in first aid management**	
5-10 minutes (correct response)	26 (23.85)
**Knowledge on part of nose to be pinched with fingers**	
Cartilage (correct response)	56 (51.38)
**Knowledge on measures to be taken for maintaining breathing during nose pinching**	
mouth breathing, avoiding blood swallowing (correct response)	56 (51.38)
**Knowledge on average time to stop bleeding after first aid management**	
10 minutes (correct response)	57 (52.29)
Knowledge on nasal decongestant used to stop bleeding(yes)	36 (33)
A ribbon gauze soaked in 2% lignocaine with adrenaline manage epistaxis (correct response)	77 (70.64)
**Average time considered to wait before referral to hospital after first aid management**	
If bleeding fails and exceeds 20 minutes even after first aid intervention (correct response)	68 (62.38)

Majority of the participants stated nose picking 81 (74.31%) followed by trauma 61 (55.96%), dry air 49 (44.95%), foreign body in nose 41 (37.61%) bleeding diathesis 40 (36.69%), postoperative condition following nasal surgery 34 (31.19%), sinusitis 34 (31.19%) and hypertension 33 (30.27%), rhinits 31 (28.44%), nasopharyngeal angiofibroma 26 (23.85%), hereditary 24 (22.02%), deviated nasal septum(DNS) 24 (22.02%), idiopathic 22 (20.18%), atheroscleosis 9 (8.33%), adenioditis 8 (7.33%), chronic renal failure(CRF) 4 (3.67%).

## DISCUSSION

We conducted a study to assess the knowledge of first semester and second semester MBBS students in a medical college of eastern Nepal regarding epistaxis and its first aid management. The mean knowledge score obtained was 11.33 out of 30, with nearly two third (62.38%) of students demonstrating above-average knowledge. We compared our findings with studies conducted in different settings, including healthcare providers, school teachers, and the general population. In Saudi Arabia, healthcare providers' knowledge scores ranged from 23% to 63%, the general population showed 50.3% high-level knowledge and among teachers it was only 15.5%.^5-9^ Contrast to our finding, every four in five medical students (89.4%) had good knowledge on epistaxis management in Tanzania.^10^ The disparities in knowledge scores may be attributed to differences in the inclusion criteria, as our study focused on fresh first and second-semester medical students. While other medical students of third fourth semesters, residents, healthcare providers working in different settings are included.^6^ We also analyzed specific components of epistaxis knowledge. Our study revealed that eighty-two (75.23%) students correctly identified the inner nasal region as the origin of epistaxis, compared to 57.6% in another study.^8^ However, 25.77% of our participants were unaware of the origin, reflecting a general lack of awareness in the population.

Participants provided varied responses on the causes of epistaxis, with digital nose picking almost three fourths (74.31%) and trauma (5.96%) being commonly mentioned. Nine participants lacked awareness of potential causes. Awareness of risk factors, such as the role of blood-thinning agents 66 (60.55%) hypertension and pregnancy 23 (20.10%), was suboptimal for primary prevention. Literature also suggest idiopathic, local, systemic, environmental and medications related causes in the occurrence of epistaxis.^2^ Though rarely occurring, hypertension is associated with indirect causes underlying vasculopathy in hypertensive patients.^2,11^ A study conducted in central Nepal identified idiopathic (38.09%) followed by hypertension (27.38%), trauma (15.47%), and coagulopathy (8.33%) as the most common risk factors of epistaxis among the patients.^12^ Similar responses were obtained from other studies conducted which stated bleeding disorders(87.1%), injury/trauma to the nose (42.3% and 81.1% respectively), hypertension(46.1% and 76.1% respectively) and nose picking(74.6%).^8,13^

Only 7 students correctly mentioned the primary management of epistaxis, highlighting the need for increased awareness of first aid measures. Contrast to this, we found relatively higher percentage of general population (40.8%) being aware of the primary measures of first aid management of epistaxis.^8^ A significantly higher percentage (71%) of medical students demonstrated the correct position and correct site for pinching the nose compared to our study.^14^ Our study found half 56 (51.38%) participants mentioning correct site(cartilage). We also found that the majority of our study participants (91.67%) who had previous experience of epistaxis had also wrong practice of primary management. The findings of our study suggest we create awareness on correct positioning technique i.e. leaning forward because adapting to a position such as leaning backward may cause complications like airway obstruction and choking. More than half 57 (52.29%) participants correctly responded that nose bleeding should be stopped within 10 minutes after first aid management and nearly two third (62.38%) correctly responded for referral to hospital if bleeding persisted even after primary intervention and lasted beyond 20 minutes in our study. Regarding the average time for nose pinching it was found that 51.9% and 81.6% participants stated correct responses as 1020 minute duration respectively.^8,13^ Compared to our study participants, significantly higher participants (72.5%) gave the correct responses on time of referral to hospital.^8^ Every four in five (88.5%) participants correctly responded right position for managing epistaxis, nearly three fourth (73.9%) responded correct site for nose pinching, two-third (67.3%) residents identified the risk factors of epistaxis.^5^ Epistaxis is frequently perceived as a non-emergency and is commonly overlooked, leading individuals to avoid seeking medical attention. Therefore, there is a critical need to raise awareness about risk management and prompt hospital referral, particularly when primary interventions prove ineffective. Neglecting epistaxis can result in life-threatening complications, especially in cases of posterior nasal bleeding, where prolonged bleeding duration and incorrect application of first aid measures may lead to severe consequences. Effective first aid interventions have the potential to manage 6570% of epistaxis cases with simple measures.^15^ Our study's strength lies in its focus on medical students, serving as a baseline for future investigations into epistaxis knowledge among a broader population. This provides an opportunity to enhance first aid management understandingnotonly among the general population but also among medical students and various healthcare professionals. By conducting the study among recently enrolled medical students, who had yet to receive theoretical knowledge or clinical practice, our results reveal pre-existing beliefs within our families, communities, and societies. This highlights prevalent cultural misconceptions and knowledge gaps in schools, families, and communities. Additionally, it underscores the importance of implementing first aid training for both medical students and the general population.

Our study comes with various limitations. Being a single-centered study with a limited sample size and a focus on younger and educated individuals may restrict the generalizability of our findings. Additionally, relying on self-reported data introduces potential biases such as social desirability or recall bias.

To enhance generalizability, we recommend conducting similar studies across diverse groups, including all categories of healthcare providers, in larger settings, and among the general population. Since our study employed a descriptive cross-sectional design, we propose that future research could benefit from an educational intervention using a pretest-posttest study design to effectively increase awareness and assess the impact of interventions.

## CONCLUSIONS

The study emphasizes the varied knowledge levels among first and second-semester medical students regarding epistaxis. While more than half demonstrated above-average understanding, targeted educational interventions are warranted. The medical curriculum should add these primary emergency care approaches.
